# *Antispila oinophylla* new species (Lepidoptera, Heliozelidae), a new North American grapevine leafminer invading Italian vineyards: taxonomy, DNA barcodes and life cycle

**DOI:** 10.3897/zookeys.170.2617

**Published:** 2012-02-22

**Authors:** Erik J. van Nieukerken, David L. Wagner, Mario Baldessari, Luca Mazzon, Gino Angeli, Vicenzo Girolami, Carlo Duso, Camiel Doorenweerd

**Affiliations:** 1 Netherlands Centre for Biodiversity, Naturalis, PO Box 9557, NL-2300 RA Leiden, The Netherlands; 2 Department Ecology & Evolutionary Biology, University of Connecticut, Storrs CT 06269–3043, USA; 3 FEM, IASMA, Center for Technology Transfer, Via E. Mach 1, I-38010, San Michele all’Adige, Trento, Italy; 4 Università di Padova, Department of Environmental Agronomy and Crop Science, AGRIPOLIS - Viale dell’Università, 16, I-35020 Legnaro (Padova), Italy

**Keywords:** Invasive species, new species, Vitaceae, viticulture, COI, leafmines, venation, genitalia, *Holocacista rivillei*, *Coptodisca*, *Antispilina*, *Phyllocnistis vitegenella*, phylogeny

## Abstract

A grapevine leafminer *Antispila oinophylla* van Nieukerken & Wagner, **sp. n.**, is described both from eastern North America (type locality: Georgia) and as a new important invader in North Italian vineyards (Trentino and Veneto Region) since 2006. The species is closely related to, and previously confused with *Antispila ampelopsifoliella* Chambers, 1874, a species feeding on Virginia creeper *Parthenocissus quinquefolia* (L.) Planchon., and both are placed in an informal *Antispila ampelopsifoliella* group. Wing pattern, genitalia, and DNA barcode data all confirm the conspecificity of native North American populations and Italian populations. COI barcodes differ by only 0–1.23%, indicating that the Italian populations are recently established from eastern North America. The new species feeds on various wild *Vitis* species in North America, on cultivated *Vitis vinifera* L. in Italy, and also on *Parthenocissus quinquefolia* in Italy. North American *Antispila* feeding on *Parthenocissus* include at least two other species, one of which is *Antispila ampelopsifoliella*. Morphology and biology of the new species are contrasted with those of North American *Antispila* Hübner, 1825 species and European *Holocacista rivillei* (Stainton, 1855). The source population of the introduction is unknown, but cases with larvae or pupae, attached to imported plants, are a likely possibility. DNA barcodes of the three European grapevine leafminers and those of all examined Heliozelidae are highly diagnostic. North American Vitaceae-feeding *Antispila* form two species complexes and include several as yet unnamed taxa. The identity of three out of the four previously described North American Vitaceae-feeding species cannot be unequivocally determined without further revision, but these are held to be different from *Antispila oinophylla*. In Italy the biology of *Antispila oinophylla* was studied in a vineyard in the Trento Province (Trentino-Alto Adige Region) in 2008 and 2009. Mature larvae overwinter inside their cases, fixed to vine trunks or training stakes. The first generation flies in June. An additional generation occurs from mid-August onwards. The impact of the pest in this vineyard was significant with more than 90% of leaves infested in mid-summer. Since the initial discovery in 2006, the pest spread to several additional Italian provinces, in 2010 the incidence of infestation was locally high in commercial vineyards. Preliminary phylogenetic analyses suggest that *Antispila* is paraphyletic, and that the *Antispila ampelopsifoliella* group is related to *Coptodisca* Walsingham, 1895, *Holocacista* Walsingham & Durrant, 1909 and *Antispilina* Hering, 1941, all of which possess reduced wing venation. Vitaceae may be the ancestral hostplant family for modern Heliozelidae.

## Introduction

There are several cases known of leafmining Lepidoptera developing into important agricultural pests, such as *Phyllocnistis citrella* Stainton, 1856 (Gracillariidae) on citrus, now a worldwide problem (Heppner and Dixon 1995) and *Leucoptera coffeella* Guérin-Méneville, 1842, *Leucoptera meyricki* Ghesquière, 1940and related species (Lyonetiidae) on coffee, that are amongst the more important coffee pests (Le Pelley 1973). Leafmining moths apparently often disperse easily, possibly due to their small size, and some have shown rapid invasions over large areas, e.g.: *Cameraria ohridella* Deschka & Dimić, 1986, *Phyllonorycter leucographella* (Zeller, 1850), *Phyllonorycter issikii* (Kumata, 1963)and *Macrosaccus robiniella* (Clemens, 1859) (Šefrová 1999; Hellrigl 2001; Šefrová 2002a, b; Šefrová and Laštůvka 2002; Davis and De Prins 2011).

Lepidopteran leafminers of grapevine (*Vitis vinifera* L.) have not yet developed into serious pests in Europe, although one North American species did recently invade European vineyards; *Phyllocnistis vitegenella* Clemens, 1859 (Lepidoptera: Gracillariidae) became established in Italy and elsewhere in Europe around 1995 (Posenato et al. 1997). The only native European leafminer of grape is *Holocacista rivillei* (Stainton, 1855) (Lepidoptera: Heliozelidae) (Hering 1957), a minor pest in vineyards in southern Europe and western Asia (see references below). *Holocacista rivillei* was described from Malta and later reported from Italy. It develops two to three generations annually (Mariani 1942; Marchi 1956; Camporese and Marchesini 1991; Dal Rì and Delaiti 1992; De Tomaso et al. 2008; Baldessari et al. 2009). Infestations leading to damage are infrequent, probably because pest populations are controlled by a complex of eulophid parasitoids (Hymenoptera) (Camporese and Marchesini 1991; Alma 1995). European populations of *Phyllocnistis vitegenella* occur in northern Italy (Marchesini et al. 2000; Villani 2002; Reggiani and Boselli 2005; Duso et al. 2011), Slovenia (Seljak 2005) and Switzerland (Cara and Jermini 2011). Itcan produce up to four generations annually and has given rise to local outbreaks in northeastern Italy (Posenato et al. 1997; Marchesini et al. 2000). The larvae of both moths produce characteristic mines in grapevine leaves; in *Holocacista rivillei* a narrow initial gallery leads subsequently to an oval full-depth blotch, from which the larva cuts out an oval pupal case or shield, in which it pupates, leaving an oval hole in the leaf. *Phyllonorycter vitegenella* makesa long tortuous gallery mine in the upper epidermis, with a distinct dark central frass line that ends in a pupal chamber. Both species can easily be detected in a vineyard based on the presence of their diagnostic leafmines.

In the summer of 2007, leafmines similar to those caused by *Holocacista rivillei* were observed in a vineyard in northeastern Italy (Borgo Valsugana, Trento province). However, the initial gallery mine was immediately enlarged into a larger blotch, indicating a different species. Adults reared from these mines differed from *Holocacista rivillei* in size and wing pattern. On their external characters they were identified as belonging to the genus *Antispila* Hübner, [1825], but not to one of the two species currently known in Europe, i.e., *Antispila treitschkiella* (Fischer von Röslerstamm, 1843) and *Antispila metallella* ([Denis & Schiffermüller], 1775) (Karsholt et al. 1995; Ellis 2010; van Nieukerken 2011) which both feed on dogwood (*Cornus* spp.). Thus, we determined that we had either an undescribed species or an alien species introduced from another continent. This pest has been reported previously as *Antispila* sp. (Baldessari et al. 2009; Duso et al. in press). Because taxonomic knowledge of the family Heliozelidae is poor, very few species being described up to modern standards, and because many of the known *Antispila* species are associated with *Vitis* species or related Vitaceae, it took some time to establish that this species was an undescribed, but common, North American species, hitherto confused with the North American *Antispila ampelopsifoliella* Chambers, 1874, described from Virginia creeper *Parthenocissus quinquefolia*. Unfortunately this confusion has already led to the incorrect introduction of the name *Antispila ampelopsifoliella* into European literature (Laštůvka 2009; van Nieukerken 2011; van Nieukerken et al. 2011a). In this paper we describe the species as *Antispila oinophylla* van Nieukerken & Wagner, sp. n., provide a diagnosis for its identification, and characterize its geographic distribution and life cycle. We also sequenced a part of the cytochrome C oxidase subunit I (COI) gene (DNA barcode) (Hebert et al. 2003b; Hebert et al. 2003a) as well as those of a selection of other Heliozelidae and the other two European grape leaf-mining micro-moths (*Holocacista rivillei* and *Phyllonorycter vitegenella*). DNA barcode data played important roles in revealing the original source of the infestation and unravelling the taxonomy of the new grape pest.

### Family Heliozelidae

The family Heliozelidae (superfamily Adeloidea) comprises 123 described species in 12 genera (van Nieukerken et al. 2011b), with the greatest diversity in North America and Australia. Larvae of the Heliozelidae produce leafmines (rarely galls) in various trees and vines, rarely herbs, and typically cut-out an oval case or shield from the leafmine, in which they moult once into a non-feeding final instar or prepupa, and finally pupate in the leaf litter or on plant parts. All are thought to overwinter in temperate regions as prepupae. Eight species of Heliozelidae occur in Europe (van Nieukerken 2011), belonging to four genera: *Antispila*, *Antispilina* Hering, 1941, *Heliozela* Herrich-Schäffer, 1853 and *Holocacista* Walsingham & Durrant, 1909. Four species of Heliozelidae were previously known from Italy: *Heliozela lithargyrellum* (Zeller, 1850), *Heliozela sericiella* (Haworth, 1828), *Holocacista rivillei* and *Antispila treitschkiella* (Fischer von Röslerstamm, 1843) (Karsholt et al. 1995), but it is likely that the fauna is incompletely sampled, and that most European species occur in Italy as well. In addition to *Antispila oinophylla*, we record here *Antispila metallella* ([Denis & Schiffermüller], 1775) as new from Italy (see Appendix B).

The grapevine family Vitaceae comprises an important group of hosts for the genus *Antispila* worldwide; out of 32 *Antispila* species for which host plants are known, 13 feed on Vitaceae, of which at least ten are associated with the genus *Vitis* (Table 1). Several more unnamed species are also associated with Vitaceae. In North America, *Heliozela aesella* Chambers, 1877 makes galls in leaves and shoots on *Vitis* (McGiffen and Neunzig 1985). There are only a few previous records of Heliozelidae as minor pests on grape: in addition to *Heliozela rivillei*, as mentioned above, *Antispila uenoi* has been recorded as a pest in Japan (Kuroko 1987; Ueno et al. 1987). *Antispila viticordifoliella* Clemens, 1860 is listed by McGiffen and Neunzig (1985) as occurring on bunch grape leaves, but not as a pest.

**Table 1.** Heliozelidae species associated with Vitaceae, type country and hostplant species. For the American species where the identity is not fully established we added [cf] between genus and species name.

**Species**	**Type country**	**Hostplants**	**source**
*Antispila oinophylla*	USA	*Vitis aestivalis*, *Vitis labrusca*, *Vitis riparia*, *Vitis vinifera*, *Vitis vulpina*, [*Parthenocissus*]	this paper
*Antispila ampelopsifoliella* Chambers, 1874	USA	*Parthenocissus quinquefolia*	Chambers 1874a, this paper
*Antispila* sp. “vitis1”	(USA)	*Vitis aestivalis*	this paper
*Antispila* [cf] *isabella* Clemens, 1860	USA	*Vitis aestivalis*, *Vitis labrusca*, *Vitis riparia*	Clemens 1860, this paper
*Antispila* sp. “vitis2”	(USA)	*Vitis aestivalis*, *Vitis riparia*	this paper
*Antispila viticordifoliella* Clemens, 1860	USA	*Vitis vulpina*	Clemens 1860, this paper
*Antispila* cf *viticordifoliella* Clemens, 1860	(USA)	*Parthenocissus quinquefolia*	this paper
*Antispila voraginella* Braun, 1927	USA	*Vitis arizonica*	Braun 1927, DLW
*Antispila ampelopsia* Kuroko, 1961	Japan	*Ampelopsis brevipedunculata*, *Vitis flexuosa*	Kuroko 1961
*Antispila inouei* Kuroko, 1987	Japan	*Vitis coignetiae*, *Vitis labruscana*	Kuroko 1987
*Antispila iviella* Kuroko, 1961	Japan	*Parthenocissus tricuspidata*	Kuroko 1961
*Antispila orbiculella* Kuroko, 1961	Japan	*Ampelopsis brevipedunculata*	Kuroko 1961
*Antispila tateshinensis* Kuroko, 1987	Japan	*Vitis coignetiae*	Kuroko 1987
*Antispila uenoi* Kuroko, 1987	Japan	*Vitis coignetiae*, *Vitis labruscana*	Kuroko 1987
*Antispila argostoma* Meyrick, 1916	India	*Cayratia trifolia*	Meyrick 1916, Fletcher 1920
*Antispila aristarcha* Meyrick, 1916	India	*Vitis* sp.	Meyrick 1916, Fletcher 1920, Fletcher 1933
*Antispila isorrhythma* Meyrick, 1926	India	*Vitis* sp.	Meyrick 1926
*Antispila* species	Indonesia, Borneo	*Leea indica*	EJvN
*Antispila* species	Australia	*Cissus antarctica*	Common 1990
*Holocacista rivillei* Stainton, 1855	Malta	*Vitis vinifera*	see text
*Heliozela aesella* Chambers, 1877	USA	*Parthenocissus quinquefolia*, *Vitis vulpina*, *Vitis* sp.	McGiffen and Neunzig 1985

## Material and methods

### Material

*Antispila oinophylla* adults were collected from Borgo Valsugana for sequencing and larvae were collected and reared for morphological studies. *Holocacista rivillei* and *Phyllocnistis vitegenella* adults were also collected from northeastern Italy (Appendix B). To obtain material of the new speciesand related species from North America for comparison, various *Antispila* mines and larvae were collected by EJvN and CDo during a field trip September-October 2010 in the states of Georgia and Tennessee and by EJvN in September 2011 (partly with DLW) in Connecticut, Massachusetts, Vermont and New York state. Other material included in the taxonomic and DNA analyses was collected by DLW, who has been collecting and rearing *Antispila* and other leafminers from across North America for three decades (Appendix B). Further material was studied or borrowed from the following collections.

Abbreviations for depositories:

ANSP Academy of Natural Sciences in Philadelphia, Pennsylvania, USA

CNC Canadian National Collection of Insects, Arachnids and Nematodes, Ottawa, Ontario, Canada

DLW Research collection of David L. Wagner, Storrs, Connecticut, USA

MCZ Museum of Comparative Zoology, Harvard University, Cambridge, Massachusetts, USA

RMNH Netherlands Centre for Biodiversity Naturalis, former Leiden Zoology collections, Leiden, Netherlands

UMDC University of Maryland, College park, USA

UPI University of Padova, Department of Environmental Agronomy and Crop Science, Italy

ZMUO Zoological Museum University of Oulu, Finland

### Rearing

Collected leaves were kept in polystyrene jars or bags, with some moss and or tissue added, until the larvae had prepared the shields. It was often necessary to remove the cut/out shields from the leaves, which were then removed from the breeding jars and dried as vouchers. Breeding jars were kept during winter in an outbuilding, and brought indoors in March, where they were kept until emergence of adults. Specimens collected during fall 2011 were still in hibernation diapause when this manuscript was accepted.

### Morphology

**Figures 1–5.**
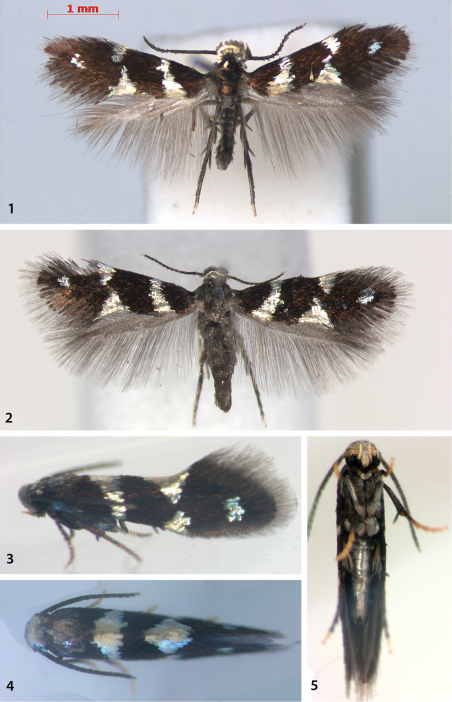
*Antispila oinophylla*, adult habitus. **1** Male holotype, RMNH.INS.24204 **2** Female paratype, RMNH.INS.24039, Italy, Borgo Valsusana. **3–5** Alive male,Georgia, paratype, emerged 29.iv.2011.

Methods for preparation of the genitalia follow Nielsen (1980a) and van Nieukerken (1985), with some minor changes. Nielsen’s unrolling technique does not work well for Heliozelidae, so we usually embedded the total genitalia in dorso-ventral position. For staining male genitalia we used (Mayers) haemaluin or phenosafranin. Wings were stained with phenosafranin and mounted in euparal. Photographs of moths, leafmines, genitalia slides and wing slides were taken with a Zeiss AxioCam digital camera attached, respectively, to a Zeiss Stemi SV11 stereo-microscope, a motorized Zeiss SteREO Discovery.V12 (only Figs 1, 40, 41) or a Zeiss Axioskop H, using Carl Zeiss AxioVision software.

The Distribution Map for North America was prepared with DMap 7.0 (Morton 2000).

### Molecular analysis

DNA was extracted destructively from larvae or adult specimens preserved in 96% or 100% ethanol or extracted in a non-destructive fashion from the abdomen of voucher specimens, which were then used to prepare genitalic dissections (protocol in Knölke et al. 2005). From some larvae used for DNA extractions, the cuticle was also cleared and saved. In Padova, total DNA was extracted applying a salting-out protocol (Patwary et al. 1994). In Leiden extractions were carried out with the Qiagen DNeasy Blood and Tissue kit (QIAGEN), using the protocol “purification of total DNA from animal tissues (spin‐column protocol).”

A 665 bp or a 658 bp fragment of the mitochondrial COI gene was amplified using the following primers: in Padua LCO1490 and HCO2198 (Folmer et al. 1994), in Leiden the Lep primers (Hebert et al. 2004), often tailed with T7 promotor and T3 tails in the shorter (amplifying 665 bp) and longer versions (amplifying 658 bp): T‐LepF1-short and T‐LepR1-short or T‐LepF1 and T‐LepR1, or when not tailed LepF1-short and LepR1-short. For some older museum specimens, the DNA was too degraded for amplifying sections over 400 bp long. For these we used internal primers (Hajibabaei et al. 2006). For details of primers see the BOLD site (http://www.barcodinglife.com/).

In Padova, amplification was carried out in 20ml volumes containing 2ml from the nucleic acid extract, 200mM dNTPs, 0.5mM of each primer, 4mM 10x PCR buffer, 2.5 mM MgCl_2_ and one unit of Taq polymerase (Promega). The reaction was performed in an INC PTC-100 thermal controller (MJ Research Inc.). Amplification conditions were as follows: the first period of denaturation was 94°C for 5 min, followed by 38 cycles of denaturation at 94°C for 1 min, annealing at 48°C for 1 min, and extension at 72°C for 1 min; the final extension cycle had a step at 72°C for 5 min. A negative control with no template was included for each series of amplifications, to detect instances of contamination. The amplified products were separated on a 1% agarose gel and visualized under UV following staining with Sybr Safe (Invitrogen). PCR products were purified with the ExoSAP-IT kit (Amersham Biosciences).

In Leiden, amplification was performed in volumes of 25 µl. The PCR cycle consisted of 3 min initial denaturation at 94°C, 15 sec cycle denaturation at 94°C, 30 sec cycle at 50°C, 40 sec cycle extension at 72°C for 40 cycles. After all cycles had finished, a final extension was performed at 72°C for 5 min. The amplified products were separated on a 1% agarose gel and visualized under UV following staining with ethidium bromide.

The sequencing at Padova was performed at the BMR Genomics Service (Padova, Italy) in an ABI PRISM automatic DNA sequencer (Applied Biosystems), in both forward and reverse direction, but for some samples only in forward direction. In Leiden PCR clean-up and sequencing was outsourced to MACROGEN on an ABI 3730XL, all samples were sequenced in both forward and reverse direction. The chromatograms were checked with Sequencher (Gene Codes Corporation) and the resulting sequences were aligned by eye in BIOEDIT 7.0.9.0 (Hall 2004).

### Tree analysis

Neighbor-joining (NJ) trees based on DNA barcode sequences of all available specimens were reconstructed with Paup* 4.0b10 (Swofford 2003). Genetic distance calculations were performed both using the Kimura two-parameter (K2P) model and uncorrected P distance (Srivathsan and Meier 2011). After initial analyses with barcodes of Italian *Phyllocnistis vitegenella*, we excluded this gracillariid from subsequent analyses (because it was so divergent from focal Heliozelidae: minimum K2P distance being greater than 18%). A Genbank sequence of *Incurvaria masculella* (Denis & Schiffermüller, 1775) (Incurvariidae), another member of the superfamily Adeloidea, was used as the outgroup. Bootstrap values were calculated with 10,000 replicates.

Phylogenetic trees based on maximum parsimony were generated with PAUP using a heuristic search, 1,000 replicates, with tree-bisection-reconnection (TBR) as the branch-swapping algorithm. A bootstrap analysis was run with TNT (Goloboff et al. 2008), a program made available with sponsorship of the Willi Hennig Society, for 10,000 replicates. From the dataset we selected one sequence for all barcode clusters with less than 2% intraspecific distance, but we included four specimens of our target species *Antispila oinophylla*, two from Italy and two from the USA.

A Bayesian Analysis was carried out with the same dataset. Model selection was performed using jModeltest 0.1.1 (Posada 2008). The best-fit model was chosen based on AIC value (Posada and Buckley 2004). Bayesian analyses were run in MrBayes 3.1.2 (Ronquist and Huelsenbeck 2003). Each analysis was run twice, starting from random starting trees, for 20 million generations and sampling every 1000 generations. Two partitioning schemes were explored: first, each codon position was given a separate partition and rate multipliers, while the second scheme combined first and second codon positions into a single partition with respect the third codon positions (Shapiro et al. 2006). Convergence of the Markov Monte Carlo chains was assessed by plotting the likelihood scores in Tracer v1.5 (Rambaut and Drummond 2007). A conservative burn-in of 5 million generations was chosen.

The sequence data generated and used in this study have been deposited in the public BOLD database (project “*Antispila* Vine introduction” [ANTVI] and GenBank (Appendix B).

### Field observations

Surveys were carried out from 2007 to 2011 to investigate the *Antispila oinophylla* distribution in northeastern Italy. We sampled commercial vineyards but also isolated vine rows and plants of Virginia creeper, *Parthenocissus quinquefolia*.

Observations on *Antispila oinophylla*phenology and behaviour were carried out in 2008 and 2009 in Borgo Valsugana (Trentino Regione). The vineyard was planted with a Chardonnay cultivar and was trained with the local “pergola” system. The vineyard received a number of fungicide treatments but insecticides were not applied. In 2008, a total of 180 leaves (30 plants, six leaves per vine) were sampled six times during the season, from May to September. In 2009, a total of 100 leaves (five replicates of ten plants, two leaves per plant) taken from the mid part of the shoots were sampled across ten dates, from May to September. In both years the number of mines produced by *Antispila oinophylla*larvae was assessed on each leaf. In 2009, active mines containing living larvae were distinguished from those vacated by the larvae (mines with larval cut-outs).

## Results

### Identification

To identify the new Italian *Antispila*, we checked all descriptions of the Vitaceae miners, as well as all other known *Antispila* species. Unfortunately, outside Europe, genitalia have been illustrated and described only for Japanese species of *Antispila*, including all five Vitaceae miners (Kuroko 1961; Kuroko 1987). For the North American fauna, only a revision for three Cornaceae-feeding *Antispila* (with genitalia illustrations), has been published (Lafontaine 1973). The genitalia of the Italian populations (Fig. 9) did not match any published illustrations. An important external character of the moths is the silver apical spot on the forewing (Figs 1–2), a feature found in just a few members of the genus, whereas the other pattern elements that we examined are more general across the genus. Similarly-sized subapical spots were only noted in descriptions of some *Antispila* from the New World, although larger subapical patches occur in Japanese species, such as *Antispila orbiculella* Kuroko, 1961. After excluding a poorly known species from Brazil as a less likely candidate, two North American Vitaceae miners with this spot were studied in more detail: *Antispila voraginella* Braun, 1927, occurring in Arizona and southern reaches of the Rocky Mountain area, and *Antispila ampelopsifoliella*, which occurs widely acrosseastern North America. The genitalia of the male holotype of *Antispila voraginella* did not match, but several specimens identified as *Antispila ampelopsifoliella* and reared from *Vitis*, had almost identical genitalia as the Italian populations. However, all specimens of *Antispila ampelopsifoliella* reared from *Parthenocissus*, were consistently different (*Antispila ampelopsifoliella* was described by Chambers from leafmines that he collected on *Parthenocissus* in Kentucky). Leafmines that we collected in 2011 in northeastern United States on *Parthenocissus* further showed that at least two species with different mines occur on that host. DNA barcoding results discussed below demonstrated that the Italian and North American examples from *Vitis* belong to the same species, and that American *Parthenocissus* feeders belong to two different barcode clusters, supporting our morphological and biological findings that two *Antispila* species, co-occurred on *Parthenocissus* in eastern North America. Material from the Chambers collection (see below) was insufficient to confirm the identity of *Antispila ampelopsifoliella*. Here we restrict the name *Antispila ampelopsifoliella* to one of the two species feeding on *Parthenocissus*. The *Vitis* miner from North America, previously misidentified in collections as being *Antispila ampelopsifoliella*, is unnamed, morphologically identical to the Italian population, and described below.

## Taxonomy

### Antispila Hübner

*Antispila* Hübner, [1825]: 419. Type species *Antispila stadtmuellerella* Hübner, [1825]: 419 (a junior synonym of *Antispila metalella* ([Denis & Schiffermüller], 1775), subsequent designation by ICZN (1988).

#### Antispila oinophylla

Van Nieukerken & Wagner sp. n.

urn:lsid:zoobank.org:act:F58A029E-A856-4414-B4EA-D7CAA6151948

http://species-id.net/wiki/Antispila_oinophylla

Figs 1 –6 9 [Fig F5] [Fig F6] –29 62 63

Antispila sp.; Baldessari et al. 2009: 68 [first record for Italy]; Duso et al. in press [pest status].
Antispila ampelopsifoliella ; Needham et al. 1928: 289 [partim]; Davis 1983: 4 [partim]; van Nieukerken 2011: Fauna Europaea database; Laštůvka 2009: S57; van Nieukerken et al. 2011a: 51. Misidentifications.
Antispila ampelopsiella ; Dyar et al. 1903: 539 [partim]; Barnes and McDunnough 1917: 181 [partim]; Forbes 1923: 226 [partim]; McDunnough 1939: 91 [partim]; Brower 1984: 29 [partim]. Misidentifications.

##### Type material.

**Holotype** ♂, **USA:** Georgia, Murray Co., Chattahoochee Nat. Forest, E of Chatsworth, GA rd 52, 523 m, 34.74066N, 84.71852W, hardwood forest along highway, leafmines on *Vitis aestivalis* var. *aestivalis*, 14.x.2010, EvN2010266, emerged 14.iv–4.v.2011, E.J. van Nieukerken & C. Doorenweerd, Genitalia slide EJvN 4204, RMNH.INS.24204 (RMNH).

##### Paratypes.

 32♂, 31♀. **Italy:** 1♂, 3♀ (all dissected), Trento, Borgo Valsusana, leafmines 2007, on *Vitis vinifera*, emerged 1.iii–26.iv.2008, M. Baldessari; 3♀, same locality, 13.viii.2008; 10♂, 1♀ (1♂ RMNH.INS.23920 dissected & DNA barcode), same locality, 18.viii.2008; 17♂, 18♀ (1♂ RMNH.INS.24038, 1♀ RMNH.INS.24039 dissected & DNA barcode), same locality, 29.vi.2009, leafmines on *Vitis vinifera*, EvN no 2009903, emerged in Leiden, 14.vii–6.viii.2009, M. Baldessari (all RMNH). **Canada:** 1♂, **Ontario**, Ottawa, mines on *Vitis*, rearing 57–112, emerged 31.iii.1958, Freeman & Lewis (CNC); 1♀, **Quebec**, Hull, mines on *Vitis*, rearing 55–228, emerged 26.vi.1956, T.N. Freeman (CNC). **USA**: 1♂, **Connecticut**, Tolland Co., Mansfield, 22.viii.1989, leafmines on Vitis, DLW89H37 breeding, emerged 4.v.1990, D.L. Wagner (DLW); 1♀ (dissected), **Connecticut**, Windham Co., Hampton, 916 Pudding Hill Rd., leafmines on *Vitis* 1–5.ix.1988, DLW 88J7, emerged 20.vii.1989, D.L. Wagner (DLW); 1♂ 1 ♀, **Georgia**, same data as holotype; 1♀, **Georgia**, Murray Co., Chattahoochee Nat. Forest, Cohutta Overlook, 730 m, 34.785356N, 84.627323W, shrub in forest clearing, leafmines on *Vitis aestivalis* var. *bicolor*, 14.x.2010, EvN2010270, emerged 19.iv.2011, E.J. van Nieukerken & C. Doorenweerd (RMNH); 1♀ (dissected, EvN 4211), **Kentucky**, [Covington], bred, [19^th^ century], Chambers, “pseudotype,” MCZ Type 1367 (MCZ); 1♂, 1♀ (♂ dissected), **Vermont**, Chittenden Co., South Burlington, leafmines on *Vitis* 11.viii.1988, DLW 88H23, emerged 30.iii–15.v.1989, D.L. Wagner (DLW).

##### Non-type material

(all in RMNH)**.** **Italy:** leafmines & larvae, Borgo Valsusana, 29.vi.2009, on *Vitis vinifera*, EvN no 2009903, M. Baldessari. **USA:** 1 larva, **Connecticut**, Tolland Co., Storrs campus, on *Vitis labrusca*, 185 m, 8.ix.2011, EvN2011168, B. Gagliardi; leafmines and larvae (being reared), **Connecticut**, New London Co., Connecticut College Arboretum, 34 m, 41.37929N, 72.11121W, on *Vitis labrusca*, 10.ix.2011, EvN2011193, E.J. van Nieukerken; leafmines and larvae (being reared), **Connecticut**, New Haven Co., West Rock Ridge SP, 125 m, 41.33353N, 72.96423W, on *Vitis aestivalis* var *aestivalis*, 10.ix.2011, EvN2011198, E.J. van Nieukerken; leafmines & larvae (DNA barcode RMNH.INS.18394), **Georgia,** same data as holotype; leafmines & larvae (DNA barcode RMNH.INS.18392), **Georgia**, Murray Co., Chattahoochee Nat. Forest, Cohutta Overlook, 730 m, 34.78535N, 84.62732W, shrub in forest clearing, leafmines on *Vitis aestivalis* var. *bicolor*, 14.x.2010, EvN2010270, E.J. van Nieukerken & C. Doorenweerd (RMNH); leafmines & 2 larvae (DNA barcode RMNH.INS.18533), **Massachusetts**, Berkshire Co., Beartown State forest, SW margin, 480 m, 42.19814N, 73.28928W, on *Vitis riparia*, 12.ix.2011, EvN2011208, E.J. van Nieukerken; leafmines & larvae (DNA barcode RMNH.INS.18558), **New York**, Essex Co., Hwy 9N, 3.5 km WSW Keeseville, 142 m, 44.49233N, 73.52042W, on *Vitis riparia*, 14.ix.2011, EvN2011237, E.J. van Nieukerken; leafmines & larvae (DNA barcode RMNH.INS.18555), **New York**, Essex Co., Wilsboro, Noblewood Park, 62 m, 44.35216N, 73.36435W, on *Vitis riparia*, 14.ix.2011, EvN2011244, E.J. van Nieukerken; leafmines & larvae (DNA barcodes RMNH.INS.18298, 18300), **Tennessee**, Blount Co., NP Great Smoky Mts, Rich Mountain Gap, 619 m, 35.64557N, 83.80537W, rich forest on limestone ridge, leafmines on *Vitis vulpina*, 2.x.2010, EvN2010119, E.J. van Nieukerken & C. Doorenweerd (RMNH); mine and larva, (DNA barcode LGSME035–06), **Tennessee**, Cocke Co., Cosby, ATBI house, 35.77771N, 83.21359W, on *Vitis* sp. 12.viii.2006, DLW 2006H55, D.L. Wagner (DLW); leafmines & larvae (being reared and DNA barcode RMNH.INS.18669), **Vermont**, Addison Co., Button Bay SP, Lake Champlain borders, 44 m, 44.18154N, 73.36892W, on *Vitis riparia*, 16.ix.2011, EvN2011253, E.J. van Nieukerken.

##### Differential diagnosis.

In North America, at least four other species have an apical silver spot (together forming the *ampelopsifoliella* group): *Antispila ampelopsifoliella* , *Antispila voraginella*, which has a darker head, an unnamed species from *Vitis* (here *Antispila* “vitis2”) and *Antispila hydrangaeella* Chambers, 1874. The latter, which is closely similar in appearance, can be separated by the greater number of white flagellomeres at the antennal tip (six segments) and feeds on *Hydrangea arborescens* L. (Hydrangeaceae). Dissection of genitalia is needed to distinguish *Antispila oinophylla* from other members of the *ampelopsifoliella* group. Male genitalia are characterised by the long carinal spine at the phallotrema and several other details; female genitalia differ by the number of cusps on the ovipositor from at least *Antispila ampelopsifoliella*.

In Europe, *Antispila oinophylla* differs from all other Heliozelidae with a similar forewing colour pattern (species of *Antispila*, *Antispilina* and *Holocacista*) by the presence of a small silvery spot in the apical part of forewing and the distinctly white head. Some Elachistidae are superficially similar, but differ in long-pointed and upcurved palpi, longer antennae and more elongate habitus.

The leafmine of *Antispila oinophylla* differs from that of *Holocacista rivillei* by its short initial gallery, which is later usually completely incorporated into the blotch, whereas the initial gallery of *Holocacista rivillei* mines is usually as long as or longer than the blotch, and remains intact. In Eastern North America other *Vitis*-feeding *Antispila* do not show the concentric arrangement of frass that is typical for *Antispila oinophylla* – particularly in thinner leaves –and the mines are often larger. Mines of *Antispila* cf *isabella* and related species are much larger, and also have much larger cut-outs, 5 mm or longer. Since not all *Vitis* miners have been comprehensively studied, mine identification cannot yet be relied on.

##### Description.

Adult (Figs 1–5). Head face and vertex covered with appressed, strongly metallic, silvery-white scales, more prominently raised in male. Palpi porrect, white; base of proboscis covered with white scales. Antenna fuscous, apical 1 or 2 flagellomeres white. Labial palp silvery white, slightly upturned. Thorax lead-coloured, shiny, contrasting with forewings. Legs grey, tarsi mostly yellowish white, especially on undersides. Forewing dark fuscous with silver-golden patterning; an outwardly oblique fascia from 1/8 of posterior margin to 1/4 of costa, narrowing towards costa; triangular (dorsal) spot at middle of posterior margin, reaching to middle of wing, smaller triangular costal spot just beyond middle, sometimes touching dorsal spot; small, silvery subapical spot in middle of wing at 3/4; fringe line distinct. Terminal fringe paler. Hindwing pale grey. Abdomen lead-coloured, including vestiture on external genitalia.

Measurements: male: forewing length 2.5–2.8 mm (2.6 ± 0.10, n=11), wingspan 5.5–6.2 mm, 25–31 antennal segments (29.1 ± 1.9, n=11); female: forewing length 2.3–2.8 mm (2.5 ± 0.16, n=10), wingspan 4.8–5.6 mm, 25–29 antennal segments (27.2 ± 1.4, n=8).

**Figures 6–7.**
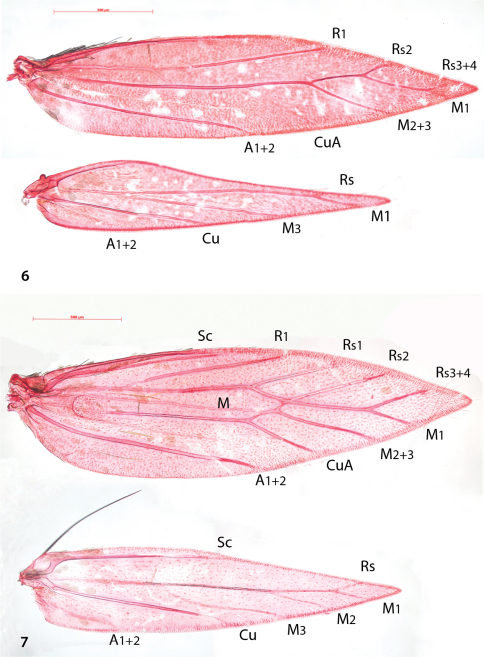
*Antispila*, venation. **6** *Antispila oinophylla*, male, Italy, RMNH.INS.24257 **7** *Antispila treitschkiella*, male, Netherlands, Leiden, RMNH.INS.24258.

Venation (Fig. 6). Forewing with Sc barely visible. R1 a separate vein, connected by persistent trachea to Rs+M stem. Rs+M terminating in five branches, interpreted as Rs2 (possibly with 1) to costa, Rs3+4 to costa just before apex, M1 to dorsum just beyond apex, M2+3 to dorsum and a weakly developed CuA. A1+2 a strong separate vein. Hindwing with Sc barely or not visible, Rs+M a strong vein, bifurcate from ca. 1/4^th^, upper vein ending in two branches: Rs and M1, lower vein single (M3); Cu and A1+2 separate veins.

**Figure 8.**
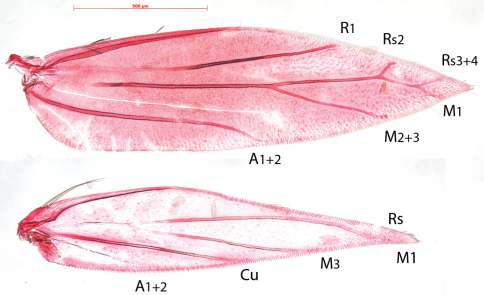
*Holocacista rivillei*, venation.Female, Italy, RMNH.INS.24259.

Compared to the complicate venation of many other *Antispila* species, including the type species *Antispila metalella*, (example in Fig. 7, *Antispila treitschkiella*) venation reduced with loss of forewing cell, separate M stem and connection between R1 and Rs, loss of Rs1 and in hindwing loss of M2. The venation more closely resembles that of *Holocacista rivillei* (Fig. 8), which is even more reduced and also lacks Cu in the forewing.

**Figures 9–16.**
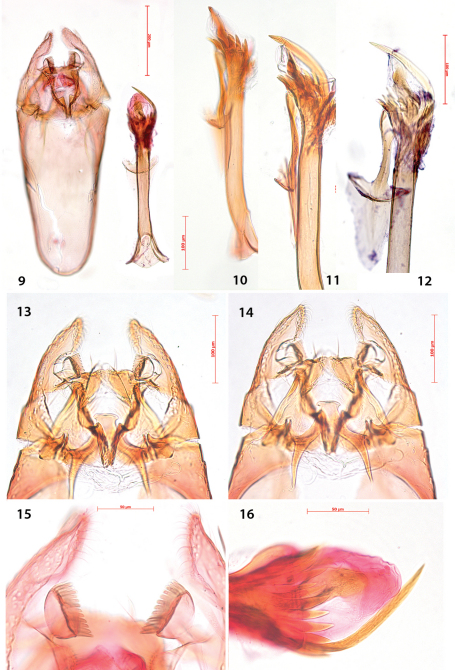
*Antispila oinophylla*, male genitalia.Paratype, Italy,RMNH.INS.23920 (**9, 15, 16**), Paratype, Italy, RMNH.INS.15247 (**12**),Holotype, RMNH.INS.24204 (**10, 11, 13–14**). **9** Complete genitalia with separate phallus in ventral view **10–12** Phallus and juxta in ventro-lateral view **15–16 **Complex of tegumen, uncus, valvae and transtilla **15** Detail of valval tips and pectinifers **16** Detail of spines near phallotrema.

Male genitalia (Figs 9–16). Uncus bar-shaped, with two large setae dorsally. Vinculum very long, anteriorly rounded, posteriorly shallowly bilobed. Valva more or less triangular, pecten on pedicel, with 10–13 comb teeth (Fig. 15); inner margin of valva with setose lobe anterior to pecten pedicel; basally with a triangular protuberance, almost touching that of other valva; transtilla with trapezoid medial plate, sublateral processes relatively short. Juxta anteriorly spade-shaped, about half as long as phallus. Phallus long, anteriorly much widened, at phallotrema with a comb of about 10–12 strong teeth and at left side a very long curved process (Figs 10–12, 16).

**Figures 17–20. F5:**
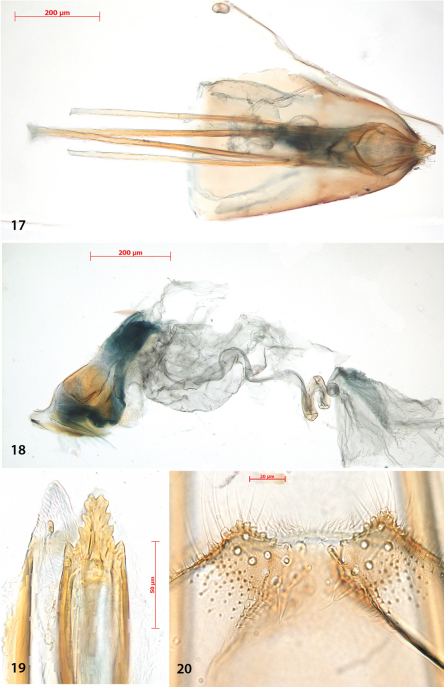
*Antispila oinophylla*, female genitalia. **17** Terminal segmentsandapophyses, ventral view, paratype, EJvN4211,USA, Kentucky (pseudotype *ampelopsifoliella*) **18** Internal genitalia, lateral view, showing sclerotisation in vestibulum,paratype, EJvN4206,USA, Connecticut **19** Ovipositor tip, dorsal view, EJvN4206 **20** Detail of S8, ventral view,paratype, Italy,RMNH.INS.15244.

Female genitalia (Figs 17–20). Ovipositor with 4–5 cusps at either side (Fig. 19). S8 medially indented, with many papillate setal sockets. Vestibulum with broad, indistinct sclerotization and no spines (Fig. 18).

##### Biology.

Host plants.In North America reared from or found as larva on summer grape *Vitis aestivalis* Michx., both var. *aestivalis* and var. *bicolor* Deam, fox grape *Vitis labrusca* L., riverbank grape *Vitis riparia* Michx. and frost grape *Vitis vulpina* L. Literature records of *Antispila* “*ampelopsifoliella*” from *Vitis* or grape likely refer to this species (Chambers 1874a, b; Forbes 1923; Needham et al. 1928). We did not find any reports of this species occurring in vineyards in North America. In Italy mines produced by *Antispila oinophylla* were detected on various *Vitis vinifera* cultivars, hybrids (e.g. *Vitis riparia* x *rupestris*) and French-American grapes (e.g. Clinton). Infestation levels on the latter were comparable with those observed on commercial vineyards. A preference for some grape cultivars (e.g. Cabernet Sauvignon, Chardonnay, Muscat) is suggested from observations carried out in mixed cultivar vineyards. It is interesting that we also found active mines on Virginia creeper *Parthenocissus quinquefolia* in Italy (Levico and Caldonazzo, Trento province) (identification of larvae confirmed by DNA barcodes, no rearing attempted), whereas we have as yet no records of *Antispila oinophylla* from this host in North America.

**Figures 21–28. F6:**
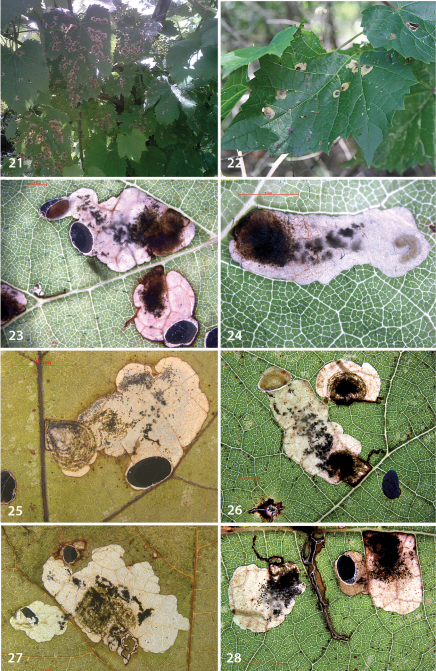
*Antispila oinophylla*, life history: leafmines on several species of *Vitis* and different localities. **21, 23, 24** Italy, Borgo Valsusana, *Vitis vinifera*, 25.vi.2009 **22** USA: Vermont, Button Bay SP, *Vitis riparia* 16.ix.2011 **25** USA:Tennessee, NP Great Smoky Mts,*Vitis vulpina*, 2.x.2010, mine in shade leaf **26, 28** USA: Georgia, type locality, *Vitis aestivalis* var. *aestivalis*, 14.x.2010 **27** USA: Vermont, Button Bay SP, *Vitis riparia*, 16.ix.2011, DNA barcode,RMNH.INS.18589.

Leafmines (Figs 21–28). The egg is inserted on the underside of a leaf, usually within 1–2 mm from a vein. The mine starts as a rather straight or slightly contorted gallery towards the vein, usually forms a right angle and often follows the vein for a short distance, then again turns away from the vein where it expands into a blotch. The gallery portion, of variable length, is usually later incorporated into the blotch mine. The frass is linear, usually occupies the complete mine width, but occasionally is deposited in a thin line (Fig. 27). In the blotch much of the blackish-brown frass is deposited close to the origin in semicircular concentric frass lines. This characteristic pattern is best seen in thin shade leaves (e.g., Figs 25, 26); in sun-exposed leaves the frass pattern is often obscured. The whole mine occupies as a rule an area of less than 10 × 10 mm; only in thin leaves are mines appreciably larger. The larva cuts out an elliptic case of about 3.2–4.0 mm long.

##### Distribution

**Figure 29.**
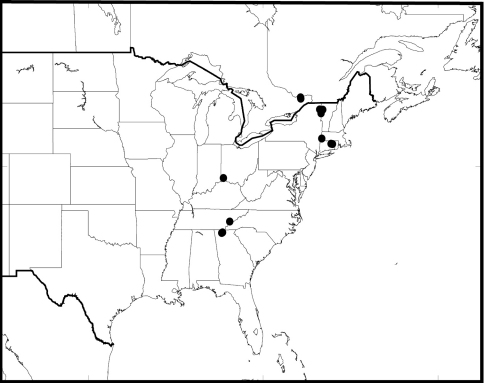
*Antispila oinophylla*, distribution in North America.

(Fig. 29, 62). In North America, *Antispila oinophylla* is known with certainty (material cited) from Canada: Ontario, Quebec; USA: Connecticut, Georgia, Kentucky, New York, Tennessee, and Vermont. Records under *Antispila ampelopsifoliella* from Maine, Missouri, and Ohio (Brower 1984, Forbes 1923) may partly refer to this species. In Europe introduced into northern Italy, see below. In our experience in the southern Appalachians and New England, at least in the fall, *Antispila oinophylla* is often the most abundant *Antispila* species occurring on *Vitis*.

##### Etymology.

The epithet *oinophylla*, a noun in apposition, is from the Greek οινος (oinos = wine) and φυλλον, plural φυλλα (phyllon, phylla = leaf), “wine leaves,” because the larva lives in the leaves of the grapevine from which wine is made.

##### Justification for status as new species.

Four species feeding on Vitaceae have been named previously from North America. No name-bearing types are available for three species, only for *Antispila voraginella* is a holotype extant. The latter is clearly different from *Antispila oinophylla*, and restricted to western North America. For the eastern species *Antispila isabella*, *Antispila viticordifoliella* and *Antispila ampelopsifoliella*, we have only the original descriptions and subsequent interpretations to establish identities. The fact that our preliminary sampling of DNA barcodes for grape-feeding *Antispila* show great diversity, complicates matters further. Below, we will discuss these three species in the chronological order of their descriptions.

*Antispila isabella* was described from mines on “Isabella grape” (a cultivar of *Vitis labrusca*) and adults (Clemens 1860). The description unequivocally describes a relatively large species without a silvery apical spot. Clemens characterizes the case (shield) as large and almost roundish – both features exclude our species. We have tentatively named one larger barcode cluster as *Antispila* cf. *isabella*, because mines and adults conform to this description.

*Antispila viticordifoliella* was also described by Clemens in 1860, from mines on “wild grapes” only, differing by a smaller case (shield) and a larva “without dots.” Although the foodplant was not explicitly mentioned by Clemens, from the species name it is evident that the host must have been *Vitis cordifolia* Michx. (a synonym of *Vitis vulpina*). In fact his very brief description could fit the mines of *Antispila oinophylla*, but subsequently the name has always (e.g. Forbes 1923) been used in the sense of Chambers (1874a), who first described the moth (as “*viticordifoliella* N. sp.?”), without an apical spot and with several, white, distal flagellomeres. He reared that moth from the same hostplant (*Vitis cordifolia*)as Clemens did, and was not able to find the mine on any other *Vitis* (Chambers 1874a: 169). One of the species that we studied from *Parthenocissus* has similar externals, and is named here *Antispila* cf *viticordifoliella* (Fig. 37). Because we haven’t been able to find or rear any similar adults from *Vitis* we are at the moment unable to establish if the *Parthenocissus* miner is indeed the same as *Antispila viticordifoliella*, but clearly it is not our species (because it lacks an apical spot). In a future revision a neotype will need to be selected to firmly anchor the identity of this species, material from the Chambers’ collection (two extant “syntypes”, see Miller and Hodges 1990) probably is most suitable for that goal. In collections and websites (e.g., http://mothphotographersgroup.msstate.edu/) the name *Antispila viticordifoliella* is often misinterpreted as the species that we call *Antispila* cf *isabella* or a closely related one.

**Figure 30.**
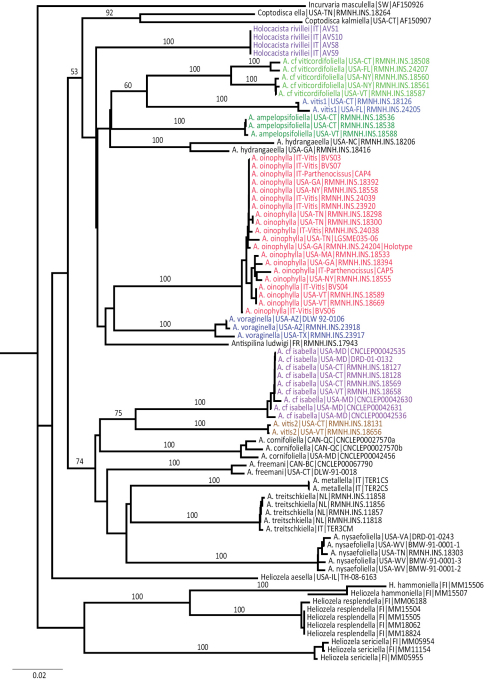
Neighbor-joining tree for heliozelid COI barcodes, based on uncorrected pairwise distances. Numbers on branches are bootstrap values, 10,000 replicates. Vitaceae-feeding clusters are coloured differently, others in black. Labels include species name or informal name, codes for country and state (in North America) and sample numbers (Genbank numbers for sequences taken from Genbank).

*Antispila ampelopsifoliella*:Chambers(1874a: 168) only briefly described the mine and larva from “*Ampelopsis quinquefolia*” [= *Parthenocissus quinquefolia*] (and stated that he “never succeeded in breeding it.”). Only a month later he described the moth under the name “*Antispila ampelopsisella*” [sic, considered as a subsequent incorrect spelling], writing: “Since that paper was placed in the hands of the Editor, many months ago, I have succeeded in rearing it from the mine [from *Parthenocissus*]” (Chambers 1874b). Theconfusionof the new specieswith *Antispila ampelopsifoliella* dates from Chambers’ original description, because he also described a moth that he reared from *Vitis* and shows the external characters of both species:

“Last summer I found its leaves [referring to a *Vitis* species] mined by a larva closely resembling that of *Antispila ampelopsifoliella*, supra, and which I suspect to be the same. ….. From it I bred the species described below, which I do not now name, as it may prove to be identical with *Antispila ampelopsifoliella*.” (Chambers 1874a). One month later he wrote: “but I believe it to be the same” (Chambers 1874b). Ever since these two publications, the species has been considered to feed both on *Parthenocissus* and *Vitis* (e.g., Forbes 1923; Brower 1984). However, our rearing and barcode data show that two or three species of *Antispila* are feeding on *Parthenocissus*, which show large barcode distances to *Antispila oinophylla* or other *Vitis* miners (Fig. 30), and thus are not identical.

In Chambers’ collection at MCZ there are three specimens under the name *Antispila ampelopsifoliella* that probably served as the basis for the adult description. These specimens were termed pseudotypes **(**Miller and Hodges 1990), since they were not available at the time of the original description, because then Chambers only had mines and larvae available. Of the three specimens, one is completely missing from the pin. The one labelled as from *Parthenocissus* unfortunately is heavily damaged, only a forewing and hindwing being present. A third specimen, a female, is complete and was dissected (Fig. 17). This specimen, however, appears to be *Antispila oinophylla*. This is no surprise, since Chambers (1874a, 1874b) considered the *Vitis* miner to be the same as the *Parthenocissus* miner, and thus he would have placed specimens reared from both hosts under the same name. There is no indication of the hostplant or the collecting year on this particular specimen, so it is useless for confirmation of the identity of *Antispila ampelopsifoliella*.

We restrict here the usage of the name *Antispila ampelopsifoliella* to the species feeding on *Parthenocissus*, with an apical spot (The generic name for *Parthenocissus quinquefolia* was *Ampelopsis* at the time Chambers described the species.) Although we have not obtained a DNA barcode form such an adult, the fact that an adult from the other cluster on this host (see below) does not have such a spot and is tentatively identified as *Antispila* cf *viticordifoliella*, we can associate *Antispila ampelopsifoliella* adults with one of the larval types. When adults are available for all barcode clusters, we suggest that a neotype be selected from material reared from *Parthenocissus* from the vicinity of Covington, Kentucky, to fix the identity of Chambers’ name.

#### DNA barcoding and species relationships

**Barcode analysis**

Neighbor-joining trees of all sequenced barcodes, both based on Kimura 2P distances and uncorrected distances give highly similar results in topology and branch lengths, we illustrate here the last one (Fig. 30). All species clusters have a bootstrap value of 100, and within-species variation is usually low or absent. We caution, however, that for several species, such as *Antispila treitschkiella* or *Holocacista rivillei* most sequences are from just one or two populations. Two species clusters show large intraspecific distances: the two specimens of *Antispila hydrangaeella* have 5.22% K2P distance and 4.99% uncorrected pairwise distance, and the species tentatively named *Antispila* cf *viticordifoliella* forms two clusters with around 4% distance in both methods. Although the mines of these clusters look superficially the same we have not studied the adults of one cluster, so it is possible that these clusters represent separate species.

We have 20 sequences representing *Antispila oinophylla*, seven of which are 100% identical, five from Italy (including one from *Parthenocissus*) and two from North America (RMNH.INS.18392 from Georgia and RMNH.INS.18558 from New York). The others are very similar, with at most five nucleotides differing from those of the core group (RMNH.INS.18394 from Georgia). The genetic distance varies from 0 to 1.23% K2P distance (1.22% uncorrected). The differences occur in 16 different positions, of which six cases are found in more than one specimen (e.g., a G instead of A in position 82 combined with a T in 316; the seven specimens forming a “clade” in Fig. 30 with RMNH.INS.18533 and BVS04; position 550: C instead of T; four specimens forming the “clade” in Fig. 30 with RMNH.INS.18533, position 634 a T instead of A in RMNH.INS.18298 and RMNH.INS.18300, both from Tennessee). Several haplotypes are found both in Italy and North America. The largest distance is between two North American specimens, one from Georgia and one from Tennessee (RMNH.INS.18394 and LGSM035–06). The genetic distance to the closest congeneric species *Antispila voraginella* is large: more than 10%.

**Phylogenetic analyses**

**Figure 31.**
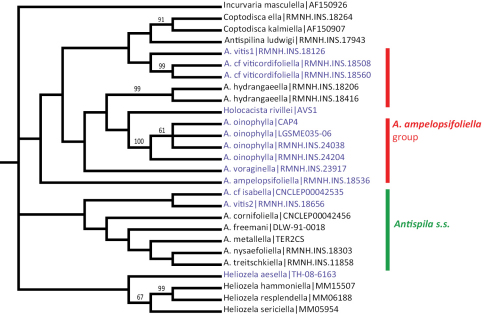
Cladogram, 50% majority rule consensus of three shortest trees from maximum parsimony analysis of COI sequences. CI = 0.361, RI = 0.456, RC = 0.168. Figures are bootstrap values from a TNT analysis (10,000 bootstrap replicates). Purple-coloured taxa are feeding on Vitaceae. The semi-strict tree differs only in the position of *Heliozela aesella* (see text).

**Figure 32.**
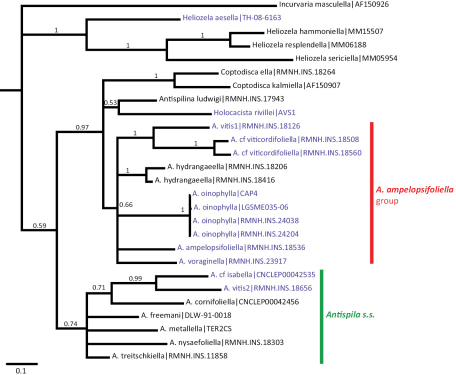
Cladogram from Bayesian analysis on three partition dataset. Figures are posterior probabilities. Purple-coloured taxa are feeding on Vitaceae.

The maximum parsimony analysis of the barcode sequences resulted in three shortest trees, of which the 50% majority rule tree is illustrated (Fig. 31). The semi-strict tree differs only in the position of *Heliozela aesella*, which forms a polytomy with the three main heliozelid clades in Figure 31. Of the 658 characters, 243 characters are parsimony informative. Bootstrap values are taken from the TNT analysis. The two Bayesian analyses of the same dataset showed few differences, we here illustrate the consensus tree based on three partitions (Fig. 32).

Both cladograms are rather similar. *Antispila oinophylla* forms a highly supported clade. Clades for *Heliozela*, a core *Antispila* grouping and a clade with several smaller genera and the *Antispila ampelopsifoliella* group were recovered, with strong support in the Bayesian analysis for thelatter clade (0.97) and for *Heliozela* (1) and less support for core *Antispila* (0.74). Within the core *Antispila* clade, the two Vitaceae species form a clade, well supported in the Bayesian tree, nested in or sister to the Cornaceae-feeding species.

The Bayesian analysis recovered a monophyletic *Antispila ampelopsifoliella* group. In both analyses this group clusters with the small genera *Coptodisca*, *Holocacista* and *Antispilina*. These all share the reduced venation as described here for *Antispila oinophylla*. Relative positions of these small genera and the two clades of *Antispila* vary amongst various analyses. In the Bayesian tree there is low support for a clade of *Antispilina* and *Holocacista*. In none of the analyses was Heliozelidae recovered as a monophyletic group.

Vitaceae-feeding taxa are indicated in the cladograms by a purple colour. If these cladograms correctly represent the phylogenetic history of the Heliozelidae, it appears that Vitaceae were the ancestral hosts for the family.

#### Comparative notes to other species

Below we will briefly treat the other Vitaceae miners amongst North American and European Heliozelidae and one other closely related species, in order to distinguish them from *Antispila oinophylla*. As there are several more *Antispila* species in North America than currently described, this is a preliminary treatment until a thorough revision can be completed. Because we have not yet been able to link some larval barcode clusters to their associated adults, the number of leafmine types described below is higher than the number of adult “species”. Material examined for each of these “taxa” is listed in the Appendix A.

##### Antispila ampelopsifoliella

Chambers

http://species-id.net/wiki/Antispila_ampelopsifoliella

Figs 35 42, 43 53 56

Antispila ampelopsifoliella Chambers, 1874a: 168. Syntypes: leafmines [USA: Kentucky, Covington] on *Ampelopsis quinquefolia* [= *Parthenocissus quinquefolia*], “pseudotypes”, Kentucky, Covington (MCZ) [examined].
Antispila ampelopsisella Chambers, 1874a: 197. Subsequent incorrect spelling.
Antispila ampelopsiella Chambers, 1874a: 198. Subsequent incorrect spelling.
Antispila ampelopsifoliella ; Needham et al. 1928: 289 [partim]; Davis 1983: 4 [partim].
Antispila ampelopsiella ; Dyar et al. 1903: 539 [partim]; Barnes and McDunnough 1917: 181 [partim]; Forbes 1923: 226; McDunnough 1939 [partim]: 91; Brower 1984: 29 [partim].

###### Differential diagnosis.

We cannotseparate *Antispila ampelopsifoliella* (Fig. 35) from *Antispila oinophylla* based on external characters: it may average a bit smaller, but our samples are too few in number to make statistical comparisons. In the male genitalia (Figs 42–43), uncus not bilobed; valva with pecten with ca. 11–13 comb spines, base of valva with rounded lobe, not triangular; juxta rather wide, with lateral groups of spines; phallus with much shorter terminal spines and a comb of rather short triangular spines near phallotrema. Female genitalia (Fig. 53): ovipositor only with 3 cusps at either side. Vestibulum with some spines.

**Figures 33–41.**
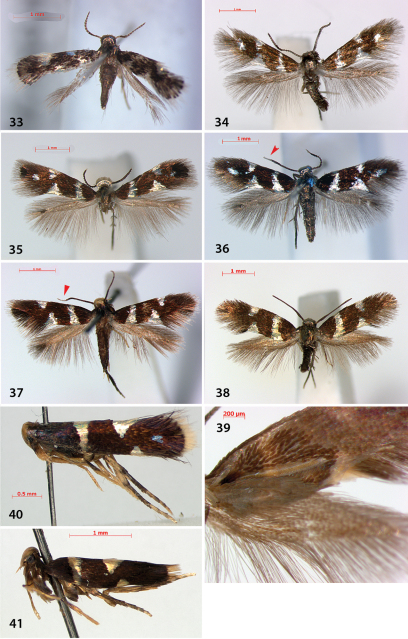
*Holocacista* and *Antispila* adult habitus in dorsal or lateral(**40, 41**)view. **33** *Holocacista rivillei*, male, Italy **34** *Antispila voraginella*, male, USA: Arizona, genitalia slide EJvN3918 **35** *Antispila ampelopsifoliella*, female, USA, Vermont: Salisbury, genitalia slide JCK15220 **36** *Antispila hydrangaeella*, female, USA: Georgia, Chattahoochee NF **37** *Antispila* cf *viticordifoliella*, female, Canada: Ottawa **38, 39** *Antispila* cf *isabella*, male, upper and underside (39) with androconial scales, USA: Connecticut, Mansfield, DLW90J8 **40** *Antispila* “vitis1”, female, USA: Florida, genitalia slide EJvN4205 **41** *Antispila* cf *viticordifoliella*, female, USA: Florida, genitalia slide EJvN4207. Arrows indicate white tipped antennae in *Antispila hydrangaeella* and cf *viticordifoliella*.

**Figures 42–47.**
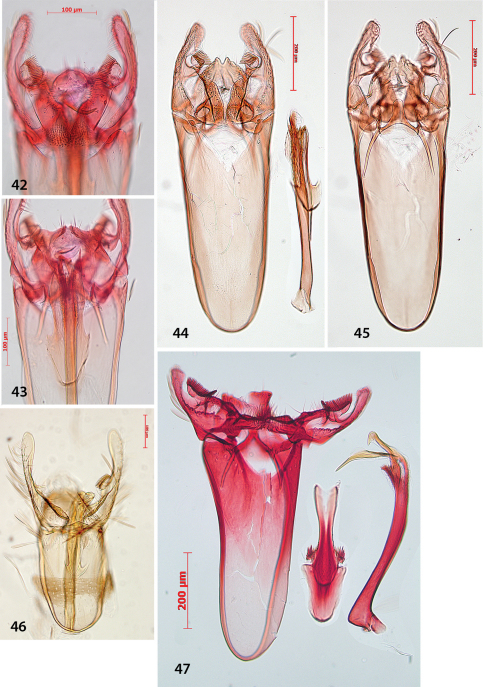
*Antispila* species, male genitalia. **42–43** *Antispila ampelopsifoliella*, USA, New York state, genitalia slide EJvN4200 **44–45** *Antispila voraginella*, USA, holotype, genitalia slide EJvN3916 **46** *Antispila* cf *isabella*, USA: Kentucky, Morehead, genitalia slide CNC MIC1859 **47** *Antispila hydrangaeella*, USA: North Carolina, NP Great Smoky Mts., genitalia slide EJvN4198.

**Figures 48–53.**
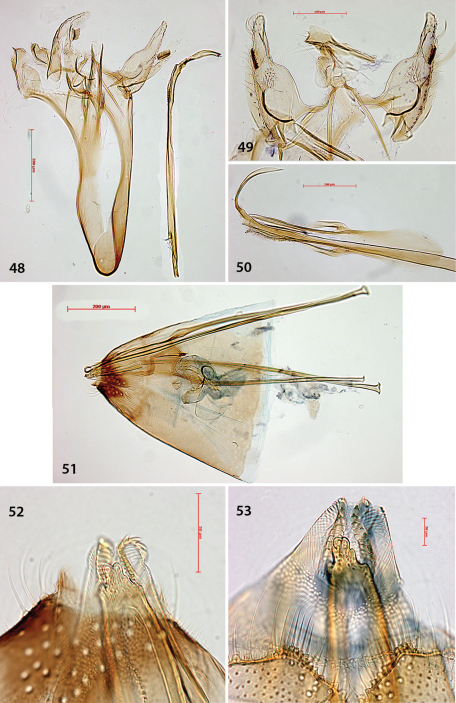
*Holocacista rivillei*, male and female genitalia, *Antispila ampelopsifoliella*, female genitalia(**53**)**. 48–50** Male genitalia, Italy, slides RMNH.INS.15248, 15250, 15251 **51–52**Female genitalia, slide RMNH.INS.15252 **53** Ovipositor and tergum 8, genitalia slide JCK15220.

###### Biology.

Hostplant: *Parthenocissus quinquefolia*.

###### Leafmines

(Fig. 56). Egg usually inserted in leaf under- or upperside close to a vein, mine starting with a relatively long contorted gallery with thin broken frass, or when it runs along margin in a straighter course, later abruptly enlarged into elongate blotch or wide gallery; frass dispersed in middle. The early narrow gallery may be as long as the elongate blotch. The mine can be found in any part of the leaf. Larva yellowish white, black head, cut-out ca 3.5–4 mm long. The mine resembles that of *Antispila hydrangaeella*. It was most frequently found in the larger and thinner ground leaves of Virginia creeper.

**Figures 54–61.**
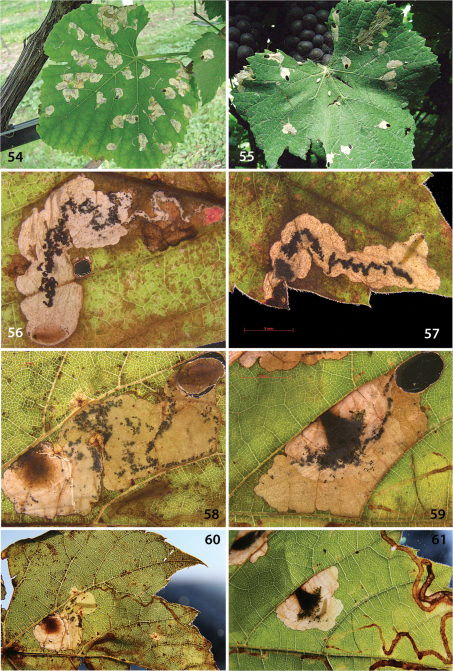
*Holocacista rivillei* and *Antispila* species, life history. **54–55** *Holocacista rivillei* on *Vitis vinifera*, Italy: Rovereto **56** *Antispila ampelopsifoliella* on *Parthenocissus quinquefolia*, USA: Button Bay SP, 16.ix.2011 **57 ***Antispila* cf *viticordifoliella* on *Phyllonorycter quinquefolia*, same locality **58, 60** *Antispila* cf *isabella* on *Vitis riparia*, USA: Button Bay SP, 16.ix.2011 **59, 61***Antispila* “vitis2” on *Vitis riparia*, samelocality. In the last four photos also parts are visible of gallery mines of *Phyllocnistis vitegenella*.

###### Distribution.

 Eastern North America, confirmed from USA: Connecticut, Kentucky, New York, Vermont and Canada: Ontario.

##### Antispila voraginella

Braun

http://species-id.net/wiki/Antispila_voraginella

Figs 34 44, 45

Antispila voraginella Braun, 1927: 191. Holotype male: **USA:** [Utah: Washington County] “B1206/Zion Canyon/Utah i.iv.9 [1926]- Antispila / voraginella / Type Braun.”, Genitalia slide EJvN 3916 [reared from mines on *Vitis arizonica*] (ANSP) [examined].

###### Differential diagnosis.

Adult (Fig. 34) very similar to and about same size as *Antispila oinophylla*, but head and thorax covered with brassy shining scales rather than silver. In male genitalia (Figs 44–45) uncus clearly bilobed, valva with fewer pecten spines: 8–10, triangular lobe absent; transtilla with narrower central plate and phallus with rather different set of spines: the long one of *oinophylla* absent, and row along phallotrema less comb-like, whereas there is a row of many spines along both sides. Female genitalia not examined.

###### Biology.

Hostplant: *Vitis arizonica*. Seems to be univoltine, larvae found in June–July northward; through September in monsoonal areas to south; adults emerging the following spring April to June.

###### Leafmines.

Mine illustrated by Powell and Opler (2009: plate 59:7). Mines rather different from those of *Antispila oinophylla*: larvae usually gregarious with mines forming large pale blotches.

###### Distribution.

 Evidently allopatric to *Antispila oinophylla* and only recorded from the Rocky Mountains: Utah, Arizona and West Texas.

### Antispila “vitis1”

Fig. 40

From this barcode cluster we have just two females from Florida (Fig. 40, one barcoded) and one larva from Connecticut, of which it is unclear to what type mine it belongs. The female is indistinguishable externally from *Antispila oinophylla*. Almost certainly this represents another new species.

#### Antispila hydrangaeella

Chambers

http://species-id.net/wiki/Antispila_hydrangaeella

Figs 36 47

Antispila hydrangaeella Chambers, 1874a: 170. Syntypes leafmines and larvae: [USA: Kentucky, Covington] on *Hydrangea arborea* [probably lost].

##### Differential diagnosis.

DNA barcodes suggest that two species might be involved, and leafmines from a population in North Carolina (Smoky Mts NP) and northern Georgia do show some differences. Described adults and larvae are from the Georgia population.Externally, adult *Antispila hydrangaeella* (Fig. 36) is extremely similar to the other species of the *Antispila ampelopsifoliella* group, but it differs by the last six antennal segments being white and by genitalia and hostplant data. In male genitalia (Fig. 47) uncus only shallowly bilobed; valva with long pecten with more comb spines: ca. 20, triangular lobe absent, at base of valva beardlike setation; juxta rather wide, with groups of spines laterally; phallus with two very long terminal spines and many small spines near phallotrema, not forming a comb. Female genitalia not examined.

##### Biology.

Hostplant: *Hydrangea arborea*.

##### Leafmines.

 One type (North Carolina) with long gallery mines, often following a vein, ending in a blotch with greenish to brown frass. The mines from Georgia with early gallery mine much contorted in a small area, with black frass, ending in elongate mine with blackish dispersed frass.

##### Distribution.

 USA: Georgia, Illinois, Kentucky, North Carolina, presumably widespread in eastern United States.

#### Antispila viticordifoliella

Clemens

http://species-id.net/wiki/Antispila_viticordifoliella

Antispila viticordifoliella Clemens, 1860: 209. Syntype mines, larva [USA: Pennsylvania, Easton], larvae on “wild grapes” [*Vitis vulpina*], August–September, Brackenridge Clemens (ANSP if extant).
Antispila viticordifoliella ; Chambers 1874a: 168 [first description of adult].

##### Differential diagnosis.

In the interpretation of this species by Chambers (1874a), as discussed above, *Antispila viticordifoliella* differs from the *Antispila ampelopsifoliella* group in missing the apical spot on the forewing and its long white antennal tip, the latter character is shared with *Antispila hydrangaeella*. We have as yet not seen such specimens originating from *Vitis*.

##### Biology.

Hostplant: *Vitis vulpina*. Leafmines not described in detail.

##### Distribution. 

USA: Kentucky, Pennsylvania. Many records are unreliable and often refer to the *isabella* complex.

#### Antispila cf viticordifoliella

Clemens

http://species-id.net/wiki/Antispila_cf_viticordifoliella

Figs 37, 41 57

##### Remarks.

 Two females (Figs 37, 41), reared from *Parthenocissus* mines, match Chambers’ (1874a) description of *Antispila viticordifoliella* adults. Because the possibility exists that two species with similar externals, feeding respectively on *Vitis* and *Parthenocissus*, are involved here, we cannot decide whether the *Parthenocissus* miner is conspecific with *viticordifoliella* or not, before we have studied genitalia and/or DNA barcodes from specimens originating from both hostplants (to date we have only barcodes from *Parthenocissus* miners and no males from either form). Moreover, there is a deep split in the barcodes from *Parthenocissus* miners, here tentatively identified as *Antispila* cf *viticordifoliella*, one cluster from New York and Vermont, the other from Connecticut and Florida. We did not see differences in mine or larva between these clusters, and thus tentatively regard them as one species.

##### Biology.

Hostplant: *Parthenocissus quinquefolia*.

##### Leafmines

(Fig. 57). Egg often inserted on leaf margin, position often hard to find, rarely near midrib, mine without a gallery at the start, an elliptic elongate blotch mine, often running along or near leaf margin; frass sometimes grouped in a clump, more typically spread in an irregular broad line. Larva yellow with almost black head, cut-out ca 3.5–4 mm long. This mine was most frequently seen in thicker leaves borne from climbing shoots.

##### Distribution.

 Canada: Ontario. USA: Connecticut, Florida, New York, Vermont.

#### Antispila cf isabella

Clemens

http://species-id.net/wiki/Antispila_cf_isabella

Figs 38, 39 46 58, 60 (59, 61 *Antispila* “vitis2”)

Antispila isabella Clemens, 1860: 209. Syntypes: [USA: Pennsylvania, Easton], larvae on “Isabella grape”, September, adults emerged May, Brackenridge Clemens (ANSP if extant).
Antispila isabella ;Chambers 1874a: 167 [redescription].

##### Differential diagnosis.

Under this name there is probably a complex of species, often with conspicuous androconial scales in males. Among the barcodes we distinguish two clusters, here tentatively named as *Antispila* cf *isabella* and *Antispila* “vitis2”. The adults described here do not necessarily belong to one of the described mine types.

Moths (Figs 38–39) of this species complex are easily distinguished from the *Antispila ampelopsifoliella* group by the missing apical spot on forewing and larger average size. Moreover males have conspicuous yellow or brown androconial scales on forewing underside (Fig. 39). The venation is also more complete (as in Fig. 7).

Male genitalia were examined of one of the species (Fig. 46) the valva is more elongate, and the pecten includes 10–13 teeth. Phallus lacks larger spines at phallotrema, but has many scale-like, small spines, and posteriorly possesses an asymmetric broad lobe; anteriorly not widened. Other individuals have not been examined; as noted above, the group is in need of revision.

##### Biology.

Hostplant: *Vitis aestivalis*, *Vitis labrusca* [incl. “Isabella” grapes], *Vitis riparia*.

##### Leafmines.

 Mines of *Antispila* cf *isabella* (Figs 58, 60) are relatively large mines, with the egg deposited near a vein. No gallery visible, mine a large blotch, with a roundish patch of reddish frass near beginning, probably attached to upper epidermis, and dispersed black frass throughout mine. Cut-out large, around 5 mm long.

Mines of *Antispila* “vitis2” (Figs 59, 61) also start on a vein, without gallery, and are relatively compact blotches, with frass concentrated in a mushroom shape or reversed triangular near beginning of mine. Cut-out large, around 4.8 mm.

##### Distribution.

 Canada: Ontario. USA: Connecticut, Georgia, Kentucky, New York, Pennsylvania, Vermont.

##### Remarks.

 Both COI sequences and external sexual secondary characters show that more species are involved. We have tentatively named the most common form as *Antispila* cf *isabella*, and research of types or material from the collections of Clemens and Chambers is needed for establishing the identities of these names.

#### Holocacista rivillei

(Stainton)

http://species-id.net/wiki/Holocacista_rivillei

Figs 8 33 48–52 54, 55

[Unnamed] Godeheu de Riville 1750: 177 [extensive description from Malta].
Alucita vitella Vallot, 1822: 253. [Preoccupied by *Alucita vitella* Fabricius, 1775].
Elachista rivillei Stainton, 1855: 87. [Malta, Godeheu de Riville, 18^th^ century, mines on *Vitis*] Renamed after Riville´s description in 1750. [types probably not existing].
Antispila rivillei ; Stainton 1869: 310 [repetition of description by Godeheu de Riville].
Antispila rivillella Rondani, 1877: 288 [Redescription, parasitoids].
Holocacista rivillei ; Walsingham and Durrant 1909: xxix [new genus, first recorded from France].

##### Differential diagnosis.

Moth (Fig. 33) much smaller than *Antispila* species, with 3.5–4 mm wingspan. Forewing pattern without apical spot, costal spots further away from wingbase than dorsal spots. Male genitalia (Figs 48–50) with slightly bilobed uncus, valva more elongate, pecten with 8–10 teeth; juxta with pair of lateral teeth; phallus extremely slender and long, ending posteriorly in long curved spine and row of small spines below that. Juxta bilobed apically. Venation reduced, rather similar to that of *Antispila oinophylla* (see Fig. 8).

##### Biology.

Hostplant: *Vitis vinifera*.

##### Leafmines

(Figs. 54–55)**.** Mine beginning with relatively long, slender gallery, later a small blotch with small cut-outs. Cocoons often attached to stems or leaves.

##### Distribution.

 Southern Europe, western and Central Asia: Spain, France, Italy, Malta, Slovenia, Croatia, Bulgaria, Greece, Ukraine, Turkey, SE Russia, Georgia, Kazakhstan, Uzbekistan, Turkmenistan (Voigt 1931; Berro 1934; Marchi 1956; Dovnar-Zapol’skij 1969; Bournier 1977; Puplesiene 1996; Maček 1999; van Nieukerken 2011).

#### Distribution of A. oinophylla in Italy (Fig. 62)

**Figure 62.**
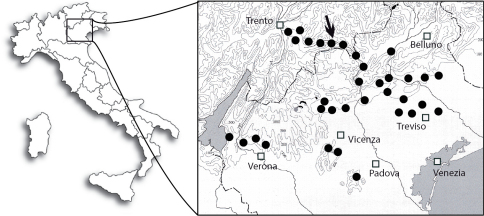
Map showing the distribution of *Antispila oinophylla* in Italy up to 2010 (filled circles = sites of occurrence; arrow = site of collection specimens for sequencing).

In Italy, *Antispila oinophylla* was detected for the first time in the summer of 2007 in a vineyard located in Valsugana (Borgo Valsugana, Trento province, Trentino-Alto Adige Region). Additional surveys conducted in the late summer of 2007 revealed its occurrence also in the neighbouring Vicenza and Belluno provinces (Veneto Region), particularly in neglected vineyards. In 2008, the distribution of the species did not differ greatly in the Trento province; elsewhere the insect was recorded in commercial vineyards of three provinces of the Veneto Region (Vicenza, Belluno and Treviso), sometimes at significant densities. In a number of vineyards *Antispila oinophylla* occurred together with *Phyllonorycter vitegenella*, rarely with *Holocacista rivillei*. In 2009 and 2010, a dense infestation was detected in commercial vineyards located in the Vicenza province (Breganze), about 80 km south of Borgo Valsugana. In this area severe symptoms had been detected as early as 2006 but they were misidentified as being caused by *Holocacista rivillei*. Since viticulture of this area is much more extensive than that around Borgo Valsugana, it is likely that *Antispila oinophylla* was introduced first in the Vicenza province and dispersed from there to the other areas. Also in 2010 the species was recorded in the Verona province, 90 km west of Breganze (E. Marchesini, pers. comm.). The distribution of *Antispila oinophylla* in Italy in 2010 is presented in Fig. 62.

#### Field observations in Italy

Observations carried out in winter 2008 showed that fully fed, final instar larvae of *Antispila oinophylla* overwintered inside their cases, fixed to the vine trunks or training stakes. Most larvae pupated in May and the first adults were seen in early June. Mines were detected first in the second half of June. Larvae of the penultimate instar cover the internal surface of the mine with a thin layer of silk, cut away an oval leaf section from both the upper and lower leaf surfaces, and then formed a case by joining the excised leaf sections with silk. Case-bearing larvae move slowly on the leaf surface and then descend with a silken thread until they contact a trunk, training stake, or other solid object to which they affix their case. In the experimental vineyard, the first cases were observed in the first half of July. An additional generation occurred from the second half of August onwards. In 2008, 86.9% of the leaves were infested with a density of 3.26 ± 0.25 (mean ± standard error) mines per leaf by the end of the first generation. In late summer, by the end of the second generation, 95.6% of leaves were infested with an average of 5.44 ± 0.37 mines per leaf.

**Figure 63.**
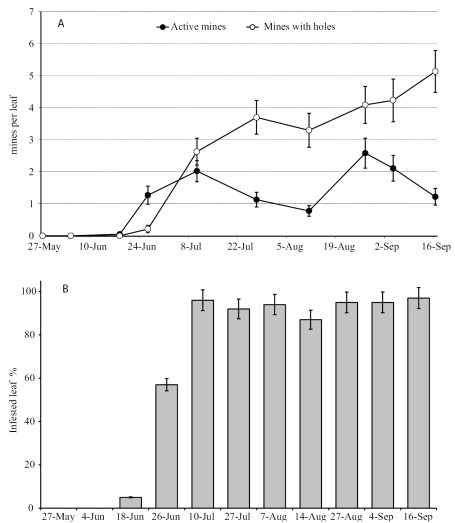
Incidence of the *Antispila oinophylla* infestation at Borgo Valsugana (Trento province, Italy) in 2009 expressed as **A** the number of mines per leaf and **B** the percentage of infested leaves (mean ± SE).

Observations carried out during 2009 in the same vineyard, confirmed the existence of two generations. Adults were detected from early June to early July. The first mines were observed in mid-June and the first cases in late June (Fig. 63). Larval densities of the first generation peaked in early July, and by late July most mines had been abandoned by the larvae. Mines of the second generation were visible beginning in the second half of August. In the first generation, 96% of leaves were infested with an average of 4.6 ± 0.53 mines per leaf. In the second generation 97% of leaves were mined with an average density of 6.67 ± 0.72 mines per leaf. Active larvae were found until mid-October.

## Discussion

### Taxonomy and identification

Identification of the unknown leafminer proved to be difficult. Many groups of Microlepidoptera remain poorly studied taxonomically. Even in North America, Powell and Opler (2009) estimated that at least one third of the microlepidopteran fauna is still undescribed. There seems to be little chance of overcoming this situation, and even groups feeding on economically important plants such as *Vitis* species, remain unstudied. Although we had assembled substantial material of *Antispila*, the morphological similarity across the genus was confusing, and only after checking several genitalia slides and COI barcodes did it become clear that what was previously called “*Antispila ampelopsifoliella*” was composed of at least two cryptic species on different hostplants. Finding COI barcode matches, in order to rule out the possibility of a non-American sibling species, took more time, because of lack of fresh material and because the *Vitis* miners in North America are more diverse than previously thought. An initial matching of the Italian pest’s barcode with that of an *Antispila* record in the BOLD identification system collected in the Great Smoky Mountains, helped focus our research efforts, and underscored the importance of a public DNA barcode reference database. In 2010 and 2011, with increased geographic and taxonomic sampling, we were able to confirm initial results and match additional sequences to those of the introduced Italian *Antispila* populations. The facts that several North American specimens show a 100% identical barcode to the majority of Italian specimens, the overall small genetic distances across all Italian specimens, and that the largest COI distance found was between two North American specimens, corroborate our position that the Italian populations represent a recent introduction from North America. All Heliozelidae species in this study differed sufficiently in their barcodes to allow reliable identification. The barcode data of North American material in addition showed us that the groups of *Vitis* and *Parthenocissus* miners are more diverse than currently recognized and that we cannot identify all taxa with certainty based strictly on morphological grounds. We also note that the North American Vitaceae-feeding *Antispila* exhibit important differences in male secondary characters and genitalia. A revision of the genus is much needed, but was not possible in the context of this study, where a name was urgently needed for a pest of grapevines. Elsewhere, for example in mainland Asia, the group of *Vitis* miners is completely unworked, and in need of taxonomic study (before new outbreaks occur).

We emphasize here the importance of combining traditional morphological descriptions with the additional dataset of DNA sequences for taxonomic groups whose identification is particularly difficult and mainly based on the description of genitalia.

An interesting observation is that we did not find any occurrence of *Antispila oinophylla* on *Parthenocissus* in its natural habitat in North America, although it utilizes that host in Italy. We found *Antispila oinophylla* mines on *Vitis* growing intertwined with *Parthenocissus* vines, that harboured two different species of *Antispila*, all occurring within a few centimetres of each other. Despite this sympatry, we did not find any indication of host shifts.

### Phylogeny

While it is generally inadvisable to rely solely on DNA barcodes for phylogenetic inferences, several recent studies suggest that some phylogenetic information could be taken from both the sequences themselves or translated amino acids (Wilson et al. 2011). Our phylogenetic results show that on the basis of the COI barcode, *Antispila* is a paraphyletic genus in relation to the genera *Holocacista*, *Antispilina* and *Coptodisca*. A generic revision of Heliozelidae has not been published, but the late Ebbe Nielsen made a primer to such a revision in his unpublished thesis (Nielsen 1980b) that has been examined by the senior author. Nielsen recognised three clades, one with *Heliozela* and some related genera, one with *Antispila* and *Antispilina* and a final one with *Ischnocanaba* Bradley, 1961 (from the Solomon Islands), *Holocacista*, *Coptodisca* and a new South American genus. The only difference with our findings is the position of *Antispilina*. Interestingly the clade of the *Antispila ampelopsifoliella* group with *Holocacista*, *Antispilina* and *Coptodisca*, as we find it, is characterised by the very similar reduced venation. A reduced venation has been reported before from some exotic *Antispila* (Kuroko 1961), but for instance all Japanese species seem to share the complex venation of the core *Antispila* as illustrated here (Kuroko 1961; Kuroko 1987). Another character noted by Nielsen to group *Holocacista* and *Coptodisca* is the habit of larvae to attach their cases to stems rather than the soil. This behavioural character is shared with *Antispila oinophylla* and other members of the *Antispila ampelopsifoliella* group. Despite the poor support for this clade on the basis of barcodes, the mitochondrial and behavioural data collectively suggest that Nielsen’s groups could be good and, should such prove to be the case, the genus *Antispila* will need to be subdivided into at least two genera. Alternatively, many of the smaller genera would need to be synonymised into one large *Antispila*, or the *Antispila ampelopsifoliella* group and the smaller genera should be combined in one genus. In the latter case, the generic name would become *Coptodisca*, which is unfortunate, since the genus in its current circumscription is well recognisable both in morphology and biology. In any case, such decisions are outside the scope of the present paper and should be made after a careful phylogenetic generic revision.

Another interesting result from our provisional phylogenetic analyses is the hypothesis that Vitaceae may form the ancestral hostplants for modern Heliozelidae. For the basal genus *Plesiozela* Karsholt & Kristensen, 2003 no host plant information is available (Karsholt and Kristensen 2003). Vitaceae occupy a rather isolated position in the angiosperm phylogeny, as sister to all core rosids (Wang et al. 2009). Other Heliozelidae feed on a wide variety of angiosperm families, but most on “eudicots”. *Heliozela* species feed mostly on rosids (Fagaceae, Betulaceae, Myrtaceae), *Antispila* species usually on asterids (Cornaceae, Rubiaceae), *Coptodisca* species on both rosid and asterid trees or shrubs (Davis 1998, van Nieukerken unpublished). Still, these results should be regarded as provisional hypotheses, and should be vigorously tested by analysing additional taxa and genes, as well as using morphological characters.

### Introduction in Italy

*Antispila oinophylla* is the first alien species of Heliozelidae introduced into Europe (Lopez-Vaamonde et al. 2010). Since our manuscript was finished a second species of Heliozelidae from North America was reported as introduction to Italy: a *Coptodisca* species on *Juglans* (Bernardo et al. 2011).

Factors leading to the introduction of *Antispila oinophylla* in Italy are unknown. *Antispila oinophylla* is the most recent Nearctic insect species reported to be damaging grapevines in Italy (first in Europe). Its invasion follows those of *Phyllocnistis vitegenella* (Posenato et al. 1997) and *Erasmoneura vulnerata* Fitch, 1851 (Hemiptera: Cicadellidae) (Duso et al. 2005). Trade of vines from North America to Italy is limited while that of the alternative host *Parthenocissus quinquefolia* seems to be more intense. However, the absence of records of *Antispila oinophylla* on *Phyllonorycter quinquefolia* in North America makes introduction with Virginia creeper a less likely pathway. Anyway, because the caterpillars routinely attach their cocoons to debris, stems or stakes, transport of *Antispila* cases is probably common, and thus not unlikely to have happened. With the frequency of modern air traffic even the transport of adults, and in particular gravid females is not impossible. The fact that *Antispila oinophylla* is an abundant and widespread species in eastern North America, together with its life history, makes such a possibility even more likely. However, it is also a warning that other species with similar life styles could be the next introduction, with an unpredictable outcome. Introduction from North America apparently occurs rather commonly; 16.5% of alien Lepidoptera species in Europe originate from North America (Lopez-Vaamonde et al. 2010).

The presence of several North American haplotypes of the DNA barcode in Italian material of *Antispila oinophylla* may indicate that the introduction could have involved more than a single introduction event.

### Infestation

Early observations, carried out during 2007 and 2008 in the Trento province, showed that the incidence of infestation by *Antispila oinophylla* was significant in vineyards not treated with insecticides. By 2009 significant infestation levels were observed in several commercial vineyards in the Trentino and Veneto Regions despite the application of insecticides. *Phyllocnistis vitegenella* is also increasingly important in commercial vineyards in northeastern Italy. Native parasitoids showed some effects in keeping *Phyllonorycter vitegenella* below economic thresholds (Marchesini et al. 2000). Local outbreaks could be associated with the use of broad-spectrum insecticides, probably because they knock out many egg and larval parasitoids, thereby disrupting the interactions between the pest and its natural enemies. Similar mechanisms could affect the relationships between *Antispila oinophylla* and its parasitoids. Knowledge of such relationships will be required to understand fully what pest status *Antispila oinophylla* might reach in the future. In Trento province, presently, the role of predators and parasitoids in controlling *Antispila oinophylla* appears to be negligible. However, in the Veneto the situation is different, with 32 to 48% of the larvae and pupae in late summer being parasitized (C. Duso and A. Pozzebon, unpublished data). The identification of parasitoids from Italian vineyards is in progress. *Phyllocnistis vitegenella* and *Holocacista rivillei* share a number of parasitoid species (Mariani 1942; Camporese and Marchesini 1991; Alma 1995; Marchesini et al. 2000). It is therefore likely that some of these will also be found to attack *Antispila oinophylla* populations.

## Supplementary Material

XML Treatment for Antispila oinophylla

XML Treatment for Antispila ampelopsifoliella

XML Treatment for Antispila voraginella

XML Treatment for Antispila hydrangaeella

XML Treatment for Antispila viticordifoliella

XML Treatment for Antispila cf viticordifoliella

XML Treatment for Antispila cf isabella

XML Treatment for Holocacista rivillei

## Appendix A

**Material studied for comparison with *Antispila oinophylla***

***Antispila ampelopsifoliella***

**Canada**: 2♂, 2♀ (2♂, 1♀ dissected), Ontario, Normandale, mines on *Parthenocissus*, rearing 57–157, emerged 16–23.iii.1958, Freeman & Lewis (CNC). **USA:** 1♂ (dissected), New York, St. Lawrence Co., Oak Point, leafmines on *Phyllonorycter quinqueguttella*, 12–17.viii.1988, DLW 88N41, emerged 14.iii.1989, D.L. Wagner (DLW); 1♀, (dissected), Vermont, Salisbury, Bryant Mtn. 16 Pudding Hill Rd., leafmines on *Phyllonorycter quinqueguttella*, 10–11.ix.1987, DLW 87J10, emerged 29.iv.1988, D.L. Wagner (DLW).

Leafmines and larvae (barcodes RMNH.INS.18536, 38), **USA:** Connecticut, Windham Co, Windham airport, Mansfield Hollow SP, 88 m, 9.ix.2011, EvN2011178, E.J. van Nieukerken & D.L. Wagner; Leafmines and larvae: New York, Essex Co, 3 km N Keene, Lacy Rd, 250 m, 14.ix.2011, EvN 2011233, E.J. van Nieukerken; Leafmines and larvae (barcode RMNH.INS.18588), Vermont, Addison Co., Button Bay SP, Lake Champlain borders, 44 m, 16.ix.2011, EvN2011254, E.J. van Nieukerken. All on*Parthenocissus quinqueguttella* and in RMNH.

***Antispila* “vitis1”**

**USA:** Larva(barcode RMNH.INS.18126), Connecticut, Tolland Co., Mansfield, 30.viii.2009, leafmines on *Vitis*, DLW 2009H285, D.L. Wagner (DLW, RMNH); 2♀ (barcode RMNH.INS.24205), Florida, Ocala Co., Ocala, Anthony, 18.vi.2006, leafmines on *Vitis aestivalis*, DLW 2006F32vii.2006, D.L. Wagner & T. Dickel (DLW).

***Antispila voraginella***

**USA:** 1♀, Arizona, Cochise Co., Huachuca Mts., Carr Canyon, Sierra Vista, *Vitis*, 4.vi.1987 [probably 1986], DLW 86F123, emerged 20.iv.1987, R. Wielgus (DLW); 1♂, Arizona, Cochise Co., Huachuca Mts., Miller canyon, *Vitis arizonica*, 31.vii.1986, DLW 86G15, emerged 23.v.1987, D.L. Wagner (DLW); 3♂, 3♀ (2♂ dissected and DNA barcode RMNH.INS.23918), Arizona, Santa Cruz Co., Sycamore Cany.S of Ruby, leafmines *Vitis arizonica*, 23.vii.1991, DLW 91G18, emerged 3.vi.1992, D.L. Wagner (DLW); 2♂, 1♀ (1♂ dissected and DNA barcode RMNH.INS.23917), Texas: Culberson Co., Guadalupe Mts NP, McKittrick canyon, leafmines *Vitis* 30–31.vii.1989, DLW 89G108, emerged 14–23.iv.1990, D.L. Wagner (DLW); 1♂ (holotype) 2♀ (paratypes), Utah: [Washington County], Zion Canyon, leafmines *Vitis arizonica*, 24.vii.[1925], B. 1206, emerged 12–14.iv.[1926], A.F. Braun (ANSP).

***Antispila hydrangaeella***

**USA:** 27♂ ♀ (1♂ dissected), leafmines & larvae (DNA barcode RMNH.INS.18416), Georgia, Gilmer Co., Chattahoochee Nat. Forest, Barnes Creek Picknick Area, 760 m, hardwood forest, leafmines on *Hydrangea arborescens*, 14.x.2010, EvN 2010279, emerged 3–18.iv.2011, E.J. van Nieukerken & C. Doorenweerd (RMNH); 29♂ ♀, leafmines & larvae (DNA barcode RMNH.INS.18206), North Carolina, Haywood Co., NP Great Smoky Mts, Big Creek area, 573 m, hardwood forest along river, leafmines on *Hydrangea arborescens*, 28.ix.2010, EvN 2010073, emerged 31.iii–9.iv.2011, E.J. van Nieukerken & C. Doorenweerd (RMNH).

***Antispila* cf *viticordifoliella***

Canada: 1♀, Ontario, Ottawa, 14.iv.1971, “Vir. creeper” [*Parthenocissus*], 70–48, G.C. Lewis (CNC). USA: Leafmines and larvae (barcode RMNH.INS.18508), **USA:** Connecticut, Windham Co, Windham airport, Mansfield Hollow SP, 88 m, leafmines on *Parthenocissus quinqueguttella*, 9.ix.2011, EvN2011178, E.J. van Nieukerken & D.L.Wagner (RMNH); 1♀ (barcode RMNH.INS.24207), Florida, Miami-dade Co., Key Biscane, Cape Florida SP, leafmines on *Parthenocissus quinqueguttella*, 19.iv.2002, DLW 2002D6, emerged 22.v.2002, R. Wagner & D.L. Wagner (DLW); Leafmines and larvae(barcodes RMNH.INS.18560–61), **USA:** New York, Essex Co, Hwy 9N, 3.5 km WSW Keeseville,142 m, 14.ix.2011, EvN 2011238, E.J. van Nieukerken; Leafmines and larvae(barcodes RMNH.INS.18587), Vermont, Addison Co., Button Bay SP, Lake Champlain borders, 44 m, 16.ix.2011, EvN2011254, E.J. van Nieukerken. All on*Parthenocissus quinqueguttella* and in RMNH.

***Antispila* cf *isabella***

**Canada:** 2♀ (dissected, MIC1862), Ontario, Simcoe, emerged 4.vi.1965, leafmines on *Vitis*, T.N. Freeman (CNC); 1♂, Ontario, Simcoe, emerged 10.iii.1971, leafmines on *Vitis*, T.N. Freeman (CNC); **USA:** 1♂, Connecticut, Windham Co., Hampton, 916 Pudding Hill Rd., leafmines on *Vitis*, 22.viii.1989, DLW 89H47, emerged 18–20.iii.1990, D.L. Wagner (DLW); Leafmines and larvae(barcodes RMNH.INS.18127–28), Connecticut, Tolland Co., Mansfield, 30.viii.2009, leafmines on *Vitis*, DLW 2009H285, D.L. Wagner (DLW, RMNH); 1♂, larvae and leafmines, Georgia, Murray Co., Chattahoochee Nat. Forest, E of Chatsworth, GA rd 52, 523 m, leafmines on *Vitis aestivalis* var. *aestivalis*, 14.x.2010, EvN2010266, emerged 14.iv–4.v.2011, E.J. van Nieukerken & C. Doorenweerd (RMNH); 1♂, (dissected, MIC1859), Kentucky, Morehead, emerged 3.vi.1963, leafmines on *Vitis*, T.N. Freeman (CNC); Leafmines and larvae(barcodes)**.** Vermont, Addison Co., Button Bay SP, Lake Champlain borders, 44 m, 44.18154N, 73.36892W, on *Vitis riparia*, 16.ix.2011, EvN2011253, E.J. van Nieukerken (RMNH); 1♂, Vermont, Chittenden Co., South Burlington, 11.viii.1988, DLW 88H23, emerged 30.iii.1989, D.L. Wagner (DLW)

***Antispila* “vitis2”**

**USA:** Leafmines and larvae(barcode RMNH.INS.18131), Connecticut, Tolland Co., Mansfield, 30.viii.2009, leafmines on *Vitis*, DLW 2009H285, D.L. Wagner (DLW, RMNH); Leafmines and larvae(barcode RMNH.INS.18656), Vermont, Addison Co., Button Bay SP, Lake Champlain borders, 44 m, leafmines on *Vitis riparia*, 16.ix.2011, EvN2011253, E.J. van Nieukerken (RMNH).

***Holocacista rivillei***

Italy: 28 adults (4♂, 1♀ dissected), Trento, Borghetto, *Vitis vinifera*, 2007, emerged i-ii.2008, M. Baldessari (RMNH).

## Appendix B

List of samples used for the DNA barcoding. (doi: 10.3897/zookeys.170.2617.app2) File format: Excel spreadsheet (xls).

**Explanation note:** List of samples used for the DNA barcoding analysis with collection site and BOLD and GenBank accession numbers. *GenBank sequences from Pellmyr and Leebens Mack (1999).

**Copyright notice:** This dataset is made available under the Open Database License (http://opendatacommons.org/licenses/odbl/1.0/). The Open Database License (ODbL) is a license agreement intended to allow users to freely share, modify, and use this Dataset while maintaining this same freedom for others, provided that the original source and author(s) are credited.

**Citation:** Nieukerken EJ, Wagner DL, Baldessari M, Mazzon L, Angeli G, Girolami V, Duso C, Doorenweerd C (2012) *Antispila oinophylla* new species (Lepidoptera, Heliozelidae), a new North American grapevine leafminer invading Italian vineyards: taxonomy, DNA barcodes and life cycle. ZooKeys 170: 29–77. doi: 10.3897/zookeys.170.2617.app2
